# Association of Life’s Crucial 9 with all-cause and cardiovascular mortality in stroke survivors and predictive value for mortality compared with Life’s Essential 8: evidence from NHANES 2005–2018

**DOI:** 10.3389/fneur.2025.1519954

**Published:** 2025-03-17

**Authors:** Xupeng Wu, Xiaofeng Li, Hong Liu

**Affiliations:** ^1^Department of Neurology, Heping Hospital Affiliated to Changzhi Medical College, Changzhi, Shanxi, China; ^2^The First Clinical Medical College, Shanxi Medical University, Taiyuan, Shanxi, China; ^3^Department of General Medicine, Linfen City People’s Hospital, Linfen, Shanxi, China

**Keywords:** cardiovascular health, Life’s Crucial 9, depression, stroke survivor, mortality

## Abstract

**Background:**

There is evidence of a positive correlation between depressive disorders and poor cardiovascular health (CVH). Recently, the inclusion of psychological health assessments into Life’s Essential 8 (LE8) has been put forward to enhance the foundation of CVH. We aimed to investigate the probable link between the innovative CVH assessment framework, Life’s Crucial 9 (LC9), and overall mortality as well as mortality associated with cardiovascular disease (CVD) among stroke survivors, while also assessing its prognostic relevance regarding mortality in comparison to LE8.

**Methods:**

This study draws on a cohort of stroke survivors identified from the National Health and Nutrition Examination Survey (NHANES), spanning survey cycles from 2005 to 2018. The LE8 was assessed by the approach recommended by the American Heart Association. The LC9 framework incorporated an additional depression score, measured by Patient Health Questionnaire-9, into the LE8 assessment. To investigate the associations between LE8 and LC9 with all-cause and cardiovascular mortality in stroke survivors, we employed multivariable Cox proportional hazards regression analyses.

**Results:**

After adjusting for covariates, each 10-point increase in LC9 was associated with a 24.5 and 30.1% reduction in all-cause and CVD mortality in stroke survivors, respectively. Participants in the highest quartile (Q4) of LC9 exhibited significantly lower mortality rates compared to those in the lowest quartile (Q1) (all-cause mortality: HR 0.412, *p* < 0.0001; CVD mortality: HR 0.327, *p* < 0.001). Similar associations were observed for LE8. Restricted cubic spline analysis indicated that both LC9 and LE8 demonstrated linearly associations with mortality post-stroke. Physical activity score, nicotine exposure score, and blood glucose score were significantly linked to all-cause and CVD mortality in stroke survivors. Adding depression score to LE8 significantly enhanced the prediction of all-cause mortality in stroke survivors (net reclassification improvement index = 9.6%, *p* = 0.033; ΔC index = 0.002, *p* = 0.0009; integrated discrimination improvement = 0.01, *p* = 0.007). The NRI of 9% (*p* = 0.086) for CVD mortality, while not statistically significant, suggests a trend toward improved classification.

**Conclusion:**

LC9 exhibited both linear and inverse correlations with all-cause and cardiovascular mortality among stroke survivors. Adding a depression score to the LE8 framework may improve the predictive accuracy for all-cause mortality in stroke survivors.

## 1 Introduction

Stroke, characterized by acute focal neurological deficits, results from various cerebrovascular causes and is primarily categorized into hemorrhagic and ischemic types (1, 2). It presents significant morbidity, mortality, and disability, profoundly affecting individuals, families, and societies (3). The Global Burden of Disease Study 2019 indicates a 70% increase in incident and an 85% rise in prevalent stroke cases over the past 30 years, with significant age-standardized incidence and prevalence rates observed in individuals under 70 (4). Over the next 30 years, stroke mortality is projected to continue to increase by 50%, and disease-adjusted life years are also projected to increase significantly (5). In the United States, the average annual medical cost per stroke patient is approximately $60,000, which is the highest of all countries (6). Despite advancements, gaps remain in current primary stroke prevention services, highlighting the urgent need to identify modifiable and practicable risk factors and foster collaborative multistakeholder efforts to implement effective stroke prevention strategies (5, 7).

Recently, the American Heart Association (AHA) updated and introduced a new tool for cardiovascular health (CVH) assessment and quantification, the Life’s Essential 8 (LE8), based on the previous Life’s Simple 7 (LS7) (8). The LE8 comprehensively evaluates eight evidence-based CVH metrics encompassing four healthy lifestyle (e.g., diet and physical activity [PA]) and four health factors (e.g., blood glucose and blood pressure), representing a new paradigm for CVH assessment (8). Since the introduction of the LE8, numerous population-based observational studies have demonstrated inverse associations between the LE8 score and various adverse health outcomes, including cardiovascular disease (CVD), chronic kidney disease, and non-alcoholic fatty liver disease (9–11). Maintaining a higher CVH has been linked to increased life expectancy and reduced risk of mortality among both men and women compared to low CVH populations (12–14). In addition, while several studies have shown an inverse relationship between the LE8 score and stroke risk, findings remain contentious (15, 16). Importantly, large population-based studies have indicated that maintaining a higher LE8 may narrow socioeconomic health inequalities (13, 17).

The bidirectional association of psychological health, including depression, with CVH is increasingly being recognized. People with CVD are at a higher risk of developing depression compared to the general population, while those with depression are also more prone to developing CVD, creating a negative feedback loop that adversely affects outcomes (18). Notably, several cross-sectional and longitudinal cohort studies have demonstrated a considerable link between CVH, as determined by the LE8 metric, and the prevalence of major depression (19, 20). Thus, in a recently published perspective, Gaffey et al. (21) suggested integrating psychological health (e.g., depression) into the existing LE8 score framework by proposing a new Life’s Crucial 9 (LC9) score. As a foundation for achieving optimal and equitable CVH, psychological health was identified as a possible underpinning for the enhancement of the existing LE8 paradigm and as an important dimension in future integrated models of cardiovascular care (21). A recent prospective cohort investigation demonstrated that LC9 was independently associated with all-cause and cardiovascular mortality among adults in U.S.; however, there was limited improvement in the predictive power of LC9 compared with LE8 for mortality (22). Available observational evidence suggests that higher LE8 is associated with reduced risk of depression and mortality after stroke (23, 24). Nevertheless, the association of LC9 with mortality in stroke survivors remains largely unknown.

This study assessed the longitudinal relationships between LC9 score and all-cause and CVD mortality in stroke survivors, as well as to elucidate whether the predictive power of the LC9 (as compared to the LE8) was improved for mortality after stroke. In summary, our study emphasizes the importance of understanding how LC9 contributes to mortality risk in stroke survivors and evaluates the necessity of incorporating depression assessments into the existing LE8 framework to enhance mortality predictions in this cohort. Given the complex interplay between CVH and mortality in stroke survivors, we conducted stratified analyses to evaluate the impact of demographic variables on these relationships. Additionally, we will evaluate the individual contributions of each LE8/LC9 component score to mortality risk, providing a more nuanced understanding of the factors driving the observed associations.

## 2 Methods

### 2.1 Study design and population

This study used the continuous NHANES database as its data source. NHANES is an ongoing, nationally representative survey that utilizes a complex, multistage probabilistic sampling technique to assess the health and nutritional status of non-institutionalized citizens in the U.S., providing extensive information on the population’s nutrition and health. The database comprises detailed questionnaire responses, physical examination results, and laboratory test data, allowing for national estimates derived from a complex multistage probability sampling design. All study procedures received approval from the National Center for Health Statistics (NCHS) Ethics Review Board, and written informed consent was obtained from all participants; data were de-identified to protect privacy. Specific details are available at https://www.cdc.gov/nchs/nhanes/index.htm.

**Figure 1:**
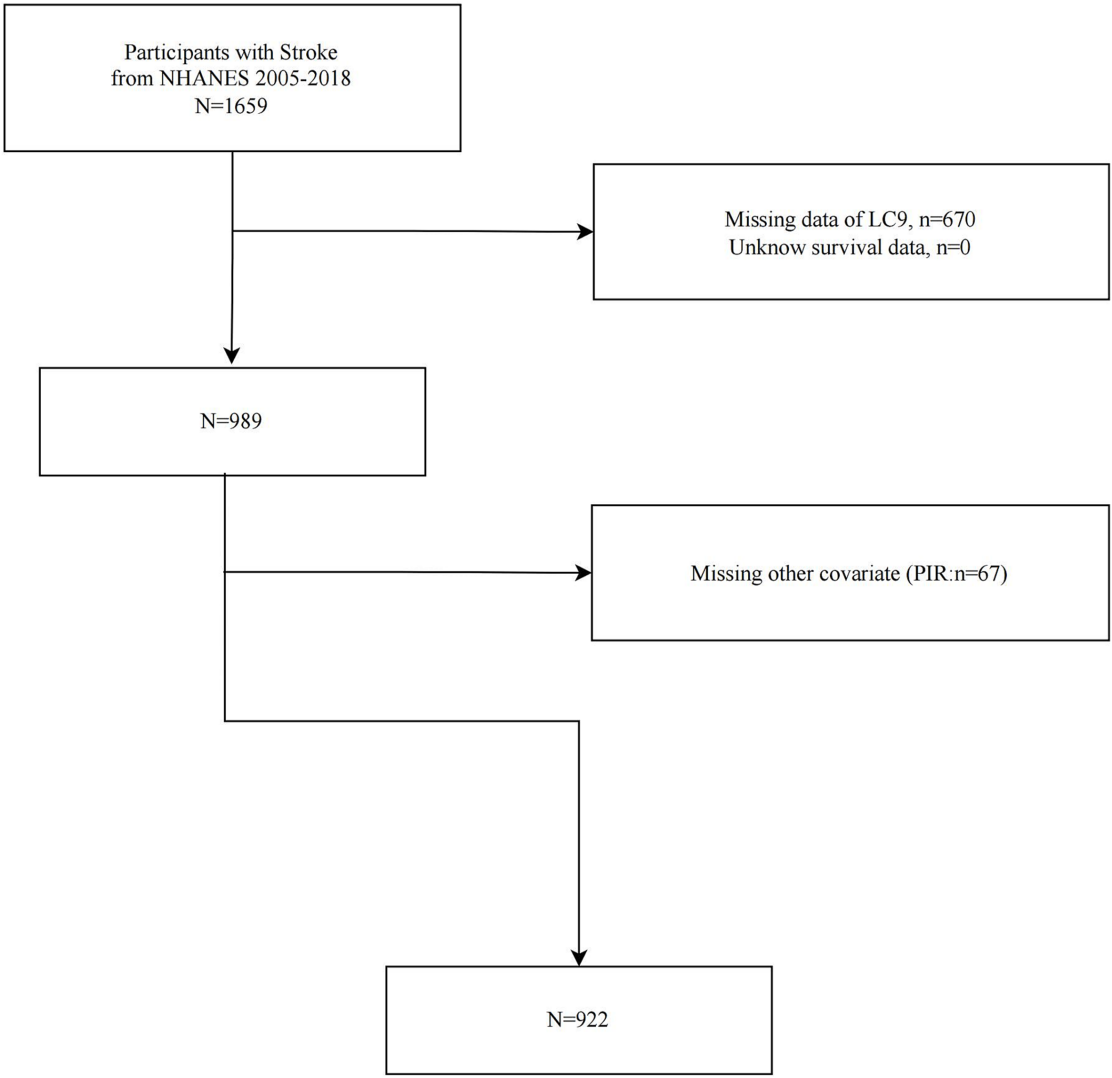
Flowchart of study population selection, NHANES 2005–2018.

In the investigation, we initially identified all 1,659 stroke survivors from NHANES 2005–2018 dataset. We then excluded 670 participants with missing LC9 data, while no participants were excluded for missing survival data (*n* = 0), and 67 participants due to incomplete covariate information. Finally, 922 eligible stroke survivors were included in further analyses. Figure 1 illustrates the process of sample selection.

### 2.2 Assessment of LC9

The LC9 score integrates the assessment of the depression score based on the LE8 (22). The assessment methods and quantitative assignment criteria for LE8 were presented in Supplementary Table S1. Briefly, the LE8 was assessed based on a composite of 8 CVH metrics, including diet, PA, nicotine exposure, sleep health, body mass index (BMI), blood lipids, blood glucose, and blood pressure (8). In this study, diet was evaluated by the Healthy Eating Index (HEI-2015), and being at a higher HEI-2015 score percentile level conferred a higher diet score. The HEI-2015 assesses adherence to a set of food groups set by the 2015–2020 Dietary Guidelines for Americans, including six food groups to be consumed in adequate amounts (fruits, vegetables, grains, dairy, protein foods, and fats) and three components to be consumed in moderate amounts (refined grains, salt, and empty calories) (25). HEI-2015 was calculated based on two 24-h dietary recalls from NHANES and United States Department of Agriculture food pattern equivalents data (11). PA (minutes of moderate or heavy PA participation per week), nicotine exposure (smoking history), sleep health (average sleep duration per night), disease history, and medication history were self-reported from standardized questionnaires in the interview portion of the NHANES (11, 14). BMI was calculated by dividing weight (kg) by height (m) assessed at the mobile examination center (MEC). Blood lipids score (non-high-density lipoprotein cholesterol, non-HDL-C) was determined by standard laboratory methods. Blood glucose score was assessed by laboratory tests (fasting blood glucose and glycosylated hemoglobin A1c) and history of diabetes. Blood pressure score was calculated from systolic and diastolic blood pressure measured at least three consecutive administrations in the MEC. Scores for each component ranged from 0 to 100, and the final LE8 score was computed based on the sum of the respective scores of the eight components divided by eight. The depression score was measured using the Patient Health Questionnaire-9 (PHQ-9). The PHQ-9 was assessed based on nine self-assessed depressive symptom items (assigned a score from 0 to 27 based on the frequency of symptoms) and has been shown to have high sensitivity and specificity for the detection of clinically significant depression (26). We calculated and categorized the depression scores according to the method of Ge et al. (22). Briefly, the depression scores were assigned 100, 75, 50, 25, and 0 points for PHQ-9 scores in the 0–4, 4–9-, 10–14-, 15–19-, and 20–27-point intervals, respectively. This approach allows the depression scores to be assigned from 0 to 100 on a scale consistent with other well-established LE8 component scores, and in a categorical manner. Finally, an individual’s LC9 score was calculated based on the sum of the scores of the eight LE8 components and the depression score divided by nine (22). Higher LC9 component scores indicate better adherence to the respective CVH metrics. LC9 were analyzed as continuous variables or categorical variables (quartiles Q1-Q4), respectively.

### 2.3 Evaluation of stroke

Participants’ stroke status was assessed based on self-reported individual interview data according to the medical conditions section (27). Participants were asked, “Has a doctor or other health professional ever told you that you had a stroke?”. Participants’ affirmative responses indicated the presence of a stroke.

### 2.4 Follow-up and mortality data

The outcomes of this study included all-cause and CVD mortality in stroke survivors. Participants were followed from baseline until death or December 31, 2019. Mortality data was collected by prospectively matching death certification records from the National Death Index database and determining factor-specific mortality through International Classification of Diseases, 10th edition codes. CVD mortality was ascertained through codes related to deaths from cardiac and cerebrovascular diseases, specifically I00–I09, I11, I13, I20–I51, and I60–I69.

### 2.5 Covariates

Based on previous research (22), key demographic variables were incorporated as prospective covariates, encompassing age (years), sex, race/ethnicity (non-Hispanic White, non-Hispanic Black, Mexican American, other Hispanic, or other race), education level (<high school education, high school education, greater than high school education), household income-poverty ratio (PIR), and marital status (non-single or single). Information on these demographic variables was obtained from self-reports on the standardized NHANES questionnaire. The PIR was evaluated according to the family’s monthly poverty level index from the income questionnaire, which is calculated based on the family’s monthly income and the federally defined poverty level (28).

### 2.6 Statistical analysis

All statistical analyses were conducted in accordance with NHANES analysis guidelines, with appropriate weighting applied. Data processing and analysis were performed using R (version 4.2.3) and EmpowerStats, with *p*-values less than 0.05 considered statistically significant. Baseline analysis was stratified by LC9 quartiles. Continuous variables were reported as mean ± standard error and tested by weighted one-way ANOVA, and categorical variables were reported as number (percentage) and analyzed by weighted chi-square test. Kaplan–Meier (KM) survival function and log-rank test were used to compare differences in all-cause and CVD-related survival over time between different quartiles of LC9. We constructed multiple multivariable Cox proportional hazards regression models to explore the association between LC9 and mortality among stroke survivors and calculated hazard ratio (HR) and 95% confidence interval (CI). Additionally, we also explored the interrelationship between the respective nine CVH metric score (continuous) of LC9 and the risk of mortality in stroke survivors. The crude models did not adjust any covariates; Model 1 adjusted for age, sex, and race/ethnicity, while Model 2 fully adjusted for age, sex, race/ethnicity, education, poverty income ratio (PIR), and marital status. To explore the potential non-linear association of LC9 score with risk of mortality in stroke survivors, we constructed restricted cubic spline (RCS) models and selected appropriate knots for smooth curve fitting. We performed stratified analyses based on included subgroups of demographic variables (age, sex, race/ethnicity, education, PIR, and marital status) and identified potential effect modifiers through interaction analyses to examine the robustness of the association of LC9 with mortality in stroke survivors across subgroups. Finally, to investigate whether the predictive value of LC9 compared with LE8 was significantly improved for the risk of mortality in stroke survivors, we calculated net reclassification improvement index (NRI), ΔC index (Harrell’s concordance index), and integrated discrimination improvement (IDI).

## 3 Results

### 3.1 Baseline characteristics

Nine hundred twenty-two stroke survivors were included with a mean age of 64.164 years and a mean LC9 score of 59.245. As LC9 quartiles increased, participants were older, had higher PIR and LC9 component scores, and a greater likelihood of being male, non-single, and possessing education beyond high school (Table 1).

**Table 1:** Baseline analysis of stroke survivors according to LC9 quartiles, NHANES 2005–2018.

Variables	Total	Q1 (*n* = 231)	Q2 (*n* = 234)	Q3 (*n* = 231)	Q4 (*n* = 226)	*p*-value
Age	64.164 ± 0.636	61.960 ± 0.869	64.871 ± 1.304	65.843 ± 1.131	64.069 ± 1.220	0.033
PIR	2.416 ± 0.074	2.032 ± 0.145	2.165 ± 0.134	2.368 ± 0.125	2.996 ± 0.136	<0.0001
LC9	59.245 ± 0.655	40.770 ± 0.567	54.267 ± 0.234	63.241 ± 0.208	75.941 ± 0.686	<0.0001
LE8	56.420 ± 0.673	38.285 ± 0.577	50.773 ± 0.341	60.062 ± 0.332	73.683 ± 0.802	<0.0001
HEI-2015 diet score	37.305 ± 1.219	20.421 ± 1.979	32.941 ± 2.288	38.885 ± 2.451	54.107 ± 2.566	<0.0001
PA score	49.323 ± 2.220	17.715 ± 3.651	35.796 ± 3.909	51.588 ± 3.648	85.884 ± 2.021	<0.0001
Nicotine exposure score	62.755 ± 1.535	40.371 ± 3.529	62.337 ± 3.118	71.184 ± 2.894	75.287 ± 2.602	<0.0001
Sleep health score	74.119 ± 1.288	58.607 ± 2.687	73.537 ± 2.467	76.369 ± 2.550	86.000 ± 1.665	<0.0001
BMI score	52.964 ± 1.505	34.368 ± 3.183	44.112 ± 2.731	59.242 ± 2.925	71.173 ± 2.436	<0.0001
Blood lipids score	60.790 ± 1.251	45.045 ± 2.447	56.390 ± 2.394	67.104 ± 2.199	72.766 ± 1.787	<0.0001
Blood glucose score	64.951 ± 1.289	50.689 ± 2.242	57.051 ± 1.926	66.752 ± 2.034	82.328 ± 2.016	<0.0001
Blood pressure score	49.152 ± 1.406	39.063 ± 2.986	44.022 ± 2.662	49.372 ± 2.617	61.923 ± 2.647	<0.0001
Depression score	81.844 ± 1.188	60.650 ± 2.740	82.215 ± 1.726	88.675 ± 2.063	94.005 ± 1.293	<0.0001
Sex						0.017
Male	450 (43.505)	90 (36.960)	122 (47.956)	112 (36.526)	126 (51.048)	
Female	472 (56.495)	141 (63.040)	112 (52.044)	119 (63.474)	100 (48.952)	
Race/ethnicity						0.141
Mexican American	78 (4.017)	14 (2.715)	18 (3.422)	22 (4.248)	24 (5.441)	
Non-Hispanic Black	244 (13.473)	65 (15.522)	71 (16.342)	62 (14.249)	46 (8.657)	
Non-Hispanic White	498 (73.700)	130 (76.339)	120 (71.455)	118 (73.583)	130 (73.446)	
Other Hispanic	51 (2.648)	14 (2.495)	16 (3.398)	11 (1.825)	10 (2.818)	
Other Race	51 (6.163)	8 (2.929)	9 (5.383)	18 (6.095)	16 (9.638)	
Marital status						0.029
Non-single	507 (62.081)	113 (56.524)	126 (56.098)	130 (63.271)	138 (70.912)	
Single	415 (37.919)	118 (43.476)	108 (43.902)	101 (36.729)	88 (29.088)	
Education						<0.001
<High school	115 (7.202)	38 (10.489)	30 (9.054)	27 (5.397)	20 (4.313)	
High school	423 (46.146)	109 (49.912)	121 (53.706)	111 (48.609)	82 (34.512)	
>High school	384 (46.651)	84 (39.599)	83 (37.240)	93 (45.994)	124 (61.175)	

Continuous variables were reported as mean ± standard error and tested by weighted one-way ANOVA, and categorical variables were reported as number (percentage) and analyzed by weighted chi-square test.

### 3.2 KM survival analysis

**Figure 2:**
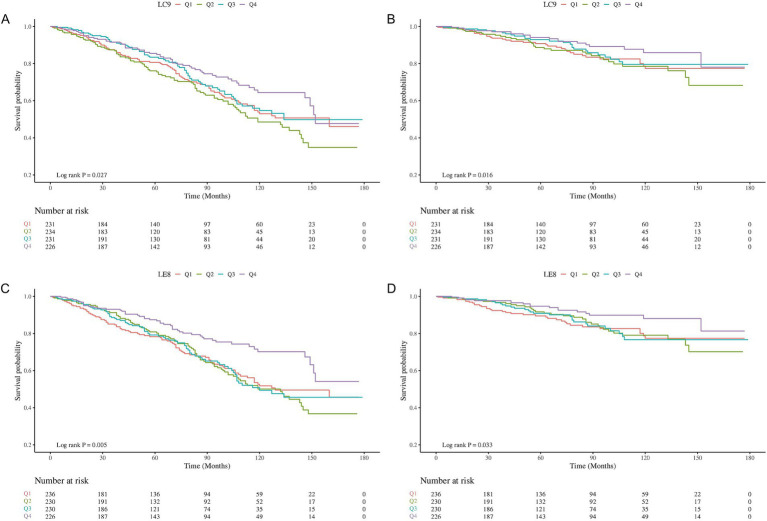
Kaplan–Meier (KM) survival analysis of LC9 and LE8 with all-cause and CVD-related survival probabilities in stroke survivors. **(A)** LC9 and all-cause mortality; **(B)** LC9 and CVD mortality; **(C)**: LE8 and all-cause mortality; **(D)** LE8 and CVD mortality.

The KM survival function demonstrated a marked increase in all-cause and CVD-related survival in stroke survivors possessing LC9 at Q4 relative to Q1 (log-rank test p of 0.027 and 0.016, respectively) (Figures 2A,B). Similarly, a higher quartile of LE8 correlated with an increased probability of all-cause (log-rank test *p* = 0.005) and CVD-related survival in stroke survivors (*p* = 0.033) (Figures 2C,D).

### 3.3 Relationship between LC9 and mortality in stroke survivors

**Figure 3:**
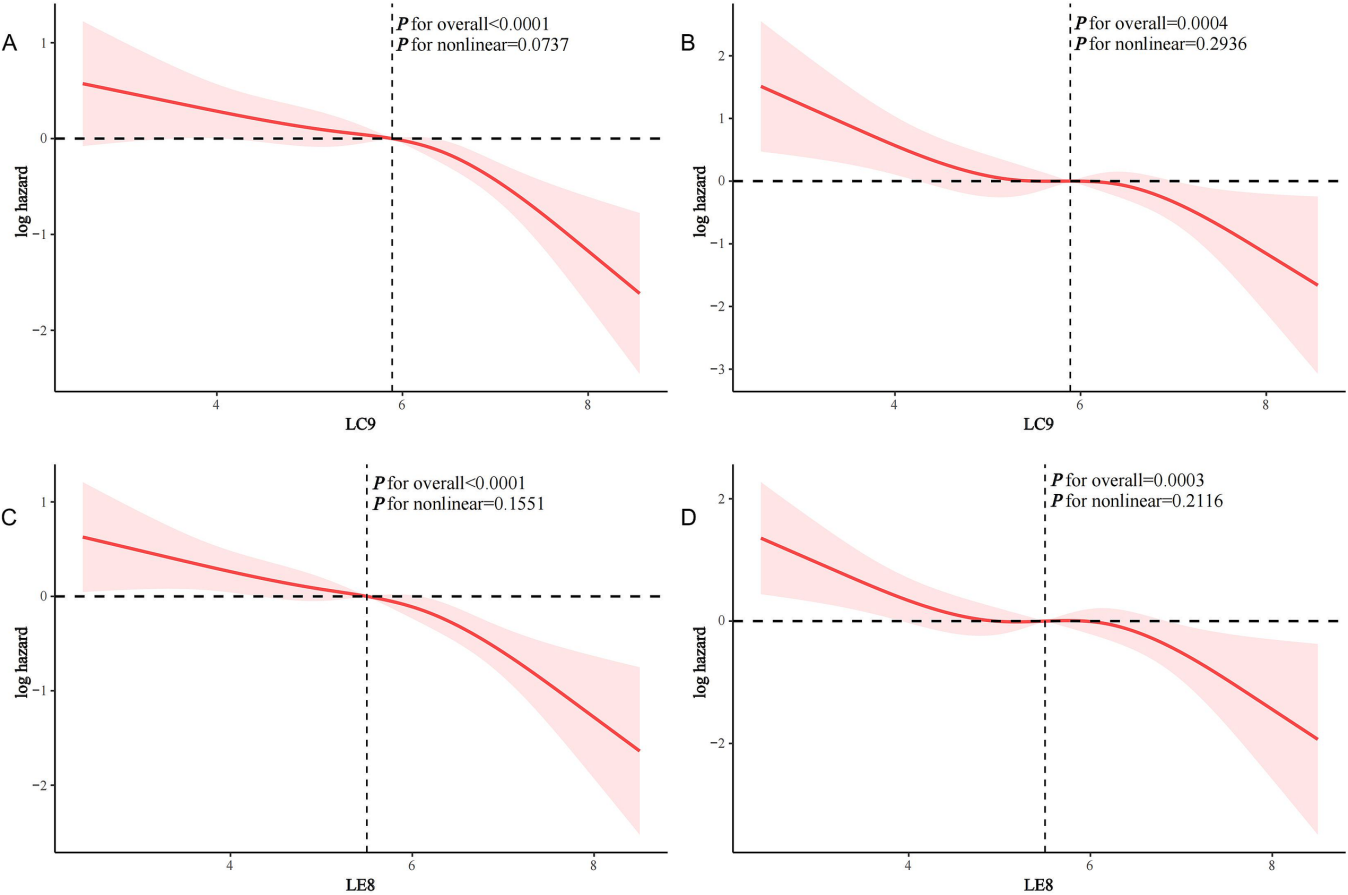
Restricted cubic spline (RCS) analysis of the association of LC9 and LE8 with all-cause and CVD mortality in stroke survivors. **(A)** LC9 and all-cause mortality; **(B)** LC9 and CVD mortality; **(C)** LE8 and all-cause mortality; **(D)** LE8 and CVD mortality.

Following a median follow-up duration of 72 months (interquartile range: 36–112), 299 stroke survivors died, of which 112 were classified as CVD related. In both the crude model and Model 1, LC9 (every 10 points) was associated with a reduced risk of all-cause mortality in stroke survivors (both *p* < 0.05). In the fully adjusted model, each 10-point increase in LC9 was associated with a 24.5% reduction in all-cause mortality in stroke survivors (HR 0.755, 95% CI 0.681–0.836, *p* < 0.0001). Participants with LC9 at Q3 and Q4 had significantly lower all-cause mortality compared to Q1 (HR 0.672 and 0.412, respectively; *p* for trend <0.0001) (Table 2). Similarly, each 10-point increase in LC9 was associated with a 30.1% reduction in CVD mortality in stroke survivors (HR 0.699, 95% CI 0.585–0.835, *p* < 0.0001). Compared to Q1, those with LC9 at Q4 had significantly lower CVD mortality (HR 0.327; *p* for trend <0.001) (Table 2). Similar patterns were found in the association of LE8 with all-cause and CVD mortality in stroke survivors (Tables 2, 3). RCS analysis showed that LC9 (continuous) was linearly associated with both all-cause and CVD mortality in stroke survivors (p for non-linearity was 0.0737 and 0.2936, respectively) (Figures 3A,B). Similarly, LE8 was linearly associated with both all-cause and CVD mortality after stroke (*p* for non-linearity 0.1551 and 0.2116, respectively) (Figures 3C,D).

**Table 2:** Association of LC9 and LE8 with all-cause mortality in stroke survivors.

ALL	Crude Model HR (95%CI)	*P*-value	Model 1 HR (95%CI)	*P*-value	Model 2 HR (95%CI)	*P*-value
LC9, per 10 points	0.859 (0.784,0.941)	0.001	0.724 (0.655,0.801)	<0.0001	0.755 (0.681,0.836)	<0.0001
LC9						
Q1	ref	ref	ref	ref	ref	ref
Q2	1.272 (0.880,1.838)	0.2	0.946 (0.710,1.261)	0.705	0.943 (0.706,1.260)	0.693
Q3	0.886 (0.583,1.346)	0.57	0.643 (0.441,0.939)	0.022	0.672 (0.464,0.974)	0.036
Q4	0.532 (0.359,0.786)	0.002	0.371 (0.260,0.529)	<0.0001	0.412 (0.292,0.581)	<0.0001
*P* for trend		<0.001		<0.0001		<0.0001
LE8, per 10 points	0.824 (0.754,0.899)	<0.0001	0.722 (0.658,0.793)	<0.0001	0.751 (0.685,0.825)	<0.0001
LE8						
Q1	ref	ref	ref	ref	ref	ref
Q2	1.017 (0.726,1.424)	0.922	0.828 (0.581,1.182)	0.299	0.846 (0.602,1.190)	0.337
Q3	1.040 (0.720,1.502)	0.836	0.667 (0.454,0.980)	0.039	0.683 (0.467,0.998)	0.049
Q4	0.407 (0.271,0.613)	<0.0001	0.328 (0.226,0.476)	<0.0001	0.368 (0.259,0.524)	<0.0001
*P* for trend		<0.0001		<0.0001		<0.0001

Crude models did not adjust for any covariates; model 1 adjusted for age, sex, and race/ethnicity; and model 2 fully adjusted for age, sex, race/ethnicity, education, PIR, and marital status.

**Table 3:** Association of LC9 and LE8 with CVD mortality in stroke survivors.

CVD	Crude model HR (95%CI)	*P*-value	Model 1 HR (95%CI)	*P*-value	Model 2 HR (95%CI)	*P*-value
LC9, per 10 points	0.845 (0.733,0.975)	0.021	0.689 (0.577,0.823)	<0.0001	0.699 (0.585,0.835)	<0.0001
LC9						
Q1	ref	ref	ref	ref	ref	ref
Q2	1.140 (0.640,2.030)	0.657	0.770 (0.475,1.249)	0.29	0.763 (0.471,1.235)	0.27
Q3	0.960 (0.484,1.905)	0.907	0.636 (0.339,1.191)	0.157	0.650 (0.348,1.214)	0.176
Q4	0.485 (0.242,0.972)	0.041	0.319 (0.165,0.615)	<0.001	0.327 (0.170,0.629)	<0.001
*P* for trend	0.811 (0.665,0.989)	0.039	0.705 (0.580,0.857)	<0.001	0.714 (0.588,0.866)	<0.001
LE8, per 10 points	0.815 (0.705,0.942)	0.005	0.697 (0.587,0.829)	<0.0001	0.709 (0.597,0.843)	<0.0001
LE8						
Q1	ref	ref	ref	ref	ref	ref
Q2	0.979 (0.555,1.729)	0.942	0.747 (0.424,1.317)	0.313	0.743 (0.421,1.314)	0.307
Q3	1.126 (0.612,2.072)	0.703	0.663 (0.366,1.200)	0.175	0.670 (0.368,1.218)	0.189
Q4	0.399 (0.188,0.848)	0.017	0.312 (0.152,0.644)	0.002	0.323 (0.157,0.663)	0.002
*P* for trend	0.798 (0.654,0.973)	0.026	0.712 (0.581,0.872)	<0.001	0.720 (0.588,0.883)	0.002

Crude models did not adjust for any covariates; model 1 adjusted for age, sex, and race/ethnicity; and model 2 fully adjusted for age, sex, race/ethnicity, education, PIR, and marital status.

### 3.4 Association of LC9 component scores with all-cause and CVD mortality in stroke survivors

**Figure 4:**
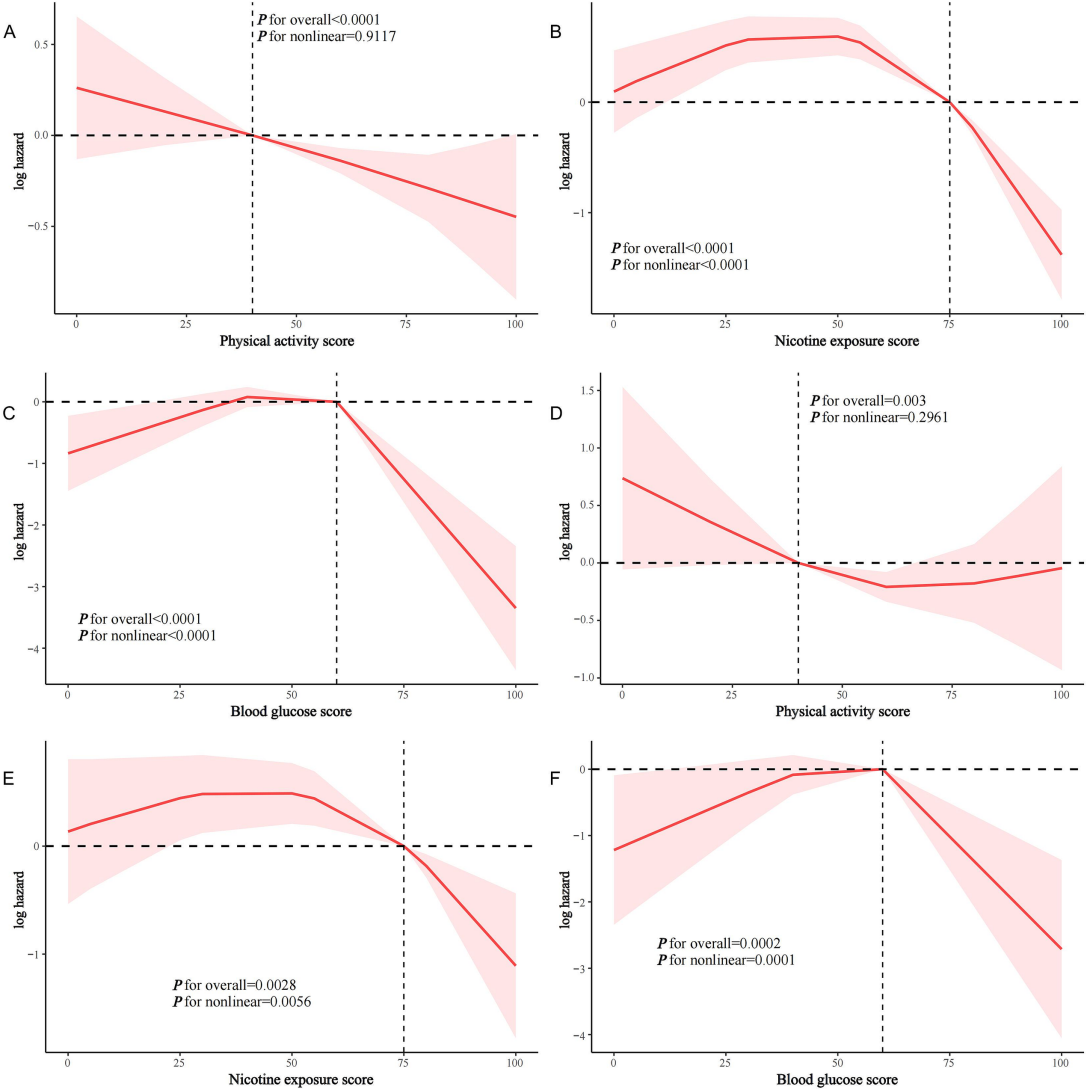
Restricted cubic spline analysis of the association of PA score, nicotine exposure score, and blood glucose score with all-cause and CVD mortality in stroke survivors. **(A)** PA score and all-cause mortality; **(B)** nicotine exposure score and all-cause mortality; **(C)** blood glucose score and all-cause mortality; **(D)** PA score and CVD mortality; **(E)** nicotine exposure score and CVD mortality; **(F)** blood glucose score and CVD mortality.

In model 2, PA score, nicotine exposure score, and blood glucose score were significantly and inversely associated with all-cause mortality in stroke survivors (HR and 95% CI per 10-point increase in score were 0.932 (0.906, 0.958), 0.909 (0.873, 0.946), and 0.839 (0.797, 0.883), respectively; all *p* < 0.0001), while none of the other component scores were not significantly associated (all *p* > 0.05) (Table 4). Similarly, only the PA score, nicotine exposure score, and blood glucose score were negatively associated with CVD mortality in stroke survivors (all *p* < 0.05) (Table 5). RCS modeling indicated that PA score was linearly associated with all-cause mortality in stroke survivors, whereas nicotine exposure score and blood glucose score were non-linearly associated (Figures 4A–C). Similar patterns were found in the association with CVD mortality (Figures 4D–F).

**Table 4:** Association of LC9 component scores (continuous) with all-cause mortality in stroke survivors.

ALL	Crude model HR (95%CI)	*P*-value	Model 1 HR (95%CI)	*P*-value	Model 2 HR (95%CI)	*P*-value
HEI-2015 diet score	1.052 (1.006,1.099)	0.025	0.968 (0.930,1.007)	0.104	0.988 (0.948,1.030)	0.566
PA score	0.900 (0.874,0.927)	<0.0001	0.923 (0.898,0.949)	<0.0001	0.932 (0.906,0.958)	<0.0001
Nicotine exposure score	0.996 (0.960,1.034)	0.841	0.891 (0.858,0.924)	<0.0001	0.909 (0.873,0.946)	<0.0001
Sleep health score	1.025 (0.982,1.069)	0.264	0.966 (0.924,1.010)	0.127	0.976 (0.933,1.021)	0.291
BMI score	1.059 (1.013,1.107)	0.012	1.010 (0.965,1.058)	0.66	1.009 (0.965,1.054)	0.703
Blood lipids score	1.001 (0.951,1.054)	0.967	0.988 (0.944,1.034)	0.6	1.003 (0.957,1.051)	0.917
Blood glucose score	0.817 (0.782,0.854)	<0.0001	0.836 (0.790,0.885)	<0.0001	0.839 (0.797,0.883)	<0.0001
Blood pressure score	0.912 (0.866,0.960)	<0.001	0.990 (0.946,1.036)	0.663	0.978 (0.934,1.024)	0.348
Depression score	1.081 (1.022,1.143)	0.006	0.977 (0.919,1.039)	0.459	0.995 (0.935,1.059)	0.877

Crude models did not adjust for any covariates; model 1 adjusted for age, sex, and race/ethnicity; and model 2 fully adjusted for age, sex, race/ethnicity, education, PIR, and marital status.

**Table 5:** Association of LC9 component scores (continuous) with CVD mortality in stroke survivors.

CVD	Crude model HR (95%CI)	*P*-value	Model 1 HR (95%CI)	*P*-value	Model 2 HR (95%CI)	*P*-value
HEI-2015 diet score	1.041 (0.979,1.107)	0.199	0.959 (0.904,1.016)	0.158	0.970 (0.918,1.024)	0.268
Physical activity score	0.892 (0.843,0.945)	<0.0001	0.916 (0.868,0.967)	0.001	0.921 (0.871,0.973)	0.003
Nicotine exposure score	1.018 (0.968,1.071)	0.489	0.910 (0.855,0.968)	0.003	0.916 (0.850,0.987)	0.021
Sleep health score	1.032 (0.974,1.093)	0.283	0.969 (0.902,1.042)	0.397	0.976 (0.909,1.048)	0.503
Body mass index score	1.035 (0.958,1.118)	0.387	0.976 (0.897,1.061)	0.57	0.974 (0.894,1.061)	0.54
Blood lipids score	0.991 (0.917,1.070)	0.811	0.956 (0.884,1.034)	0.26	0.963 (0.891,1.039)	0.33
Blood glucose score	0.837 (0.795,0.882)	<0.0001	0.868 (0.811,0.929)	<0.0001	0.872 (0.815,0.932)	<0.0001
Blood pressure score	0.896 (0.832,0.966)	0.004	0.976 (0.911,1.045)	0.481	0.966 (0.902,1.035)	0.325
Depression score	1.062 (0.996,1.132)	0.065	0.938 (0.871,1.010)	0.089	0.941 (0.869,1.018)	0.129

Crude models did not adjust for any covariates; model 1 adjusted for age, sex, and race/ethnicity; and model 2 fully adjusted for age, sex, race/ethnicity, education, PIR, and marital status.

### 3.5 Stratified analysis

**Figure 5:**
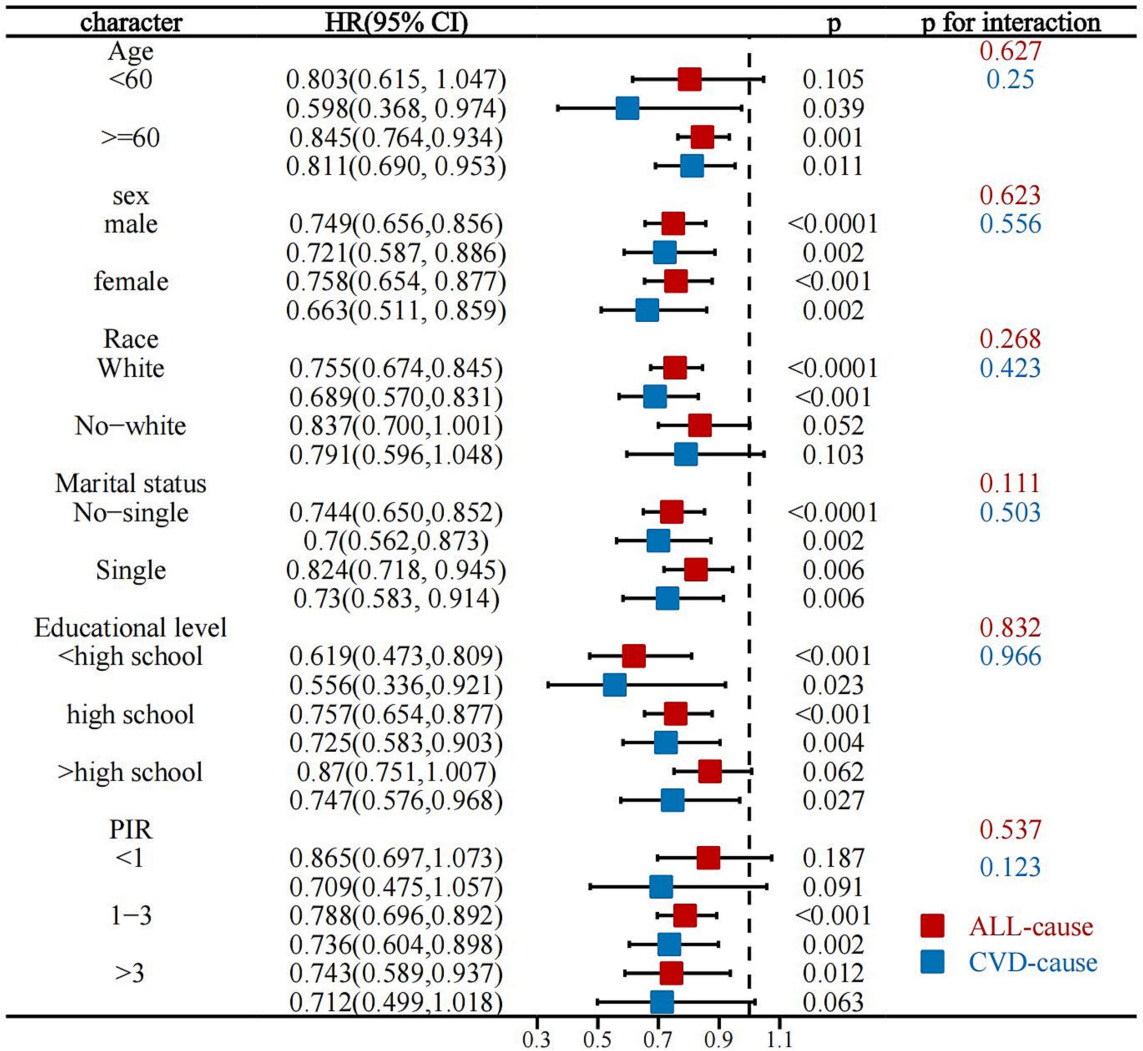
Stratified analysis of the association between LC9 and all-cause and CVD mortality in stroke survivors.

Interaction analyses showed that the association of LC9 with all-cause mortality in stroke survivors remained stable across all subgroups (*p* for interaction all >0.05). Similarly, the association of LC9 with CVD mortality in stroke survivors was not affected by any demographic variables, demonstrating the robustness of these associations (Figure 5).

### 3.6 Predictive value of LC9 compared with LE8 for all-cause and CVD mortality in stroke survivors

The addition of depression score assessment to LE8 significantly improved the predictive value of all-cause mortality in stroke survivors compared to LE8 alone (NRI = 9.6, 95% CI 0.4–19.3%, *p* = 0.033; ΔC index = 0.002, 95% CI 0.001–0.003, *p* = 0.0009; IDI = 0.01, 95% CI 0.002–0.023, *p* = 0.007). The addition of the depression score to the LE8 improved the predictive value of cardiovascular disease mortality to a similar extent as all-cause mortality (in terms of the NRI), although not statistically significant (NRI = 9%, *p* = 0.086; ΔC index = 0.001, *p* = 0.069; IDI = 0.005, *p* = 0.047) (Table 6).

**Table 6:** Predictive value of adding depression score to LE8 compared to LE8 alone for all-cause and CVD mortality in stroke survivors.

	NRI	ΔC index	IDI
All-cause			
LE8	Ref.	Ref.	Ref.
LE8 + PHQ9 score	0.0960 (0.0040, 0.1930) 0.0330	0.002 (0.001,0.003) 0.0009	0.0100 (0.0020, 0.0230) 0.0070
CVD			
LE8	Ref.	Ref.	Ref.
LE8 + PHQ9 score	0.0900(−0.0300, 0.2150) 0.0860	0.001(−0.001,0.003) 0.0690	0.0050 (0.0000, 0.0130) 0.0470

## 4 Discussion

Our investigation involved a national prospective cohort study of stroke survivors, revealing that LC9 was inversely related to the risk of all-cause and CVD mortality, with higher LC9 scores significantly linked to a reduced risk of mortality following stroke. RCS analysis showed that LC9 and LE8 were linearly associated with both all-cause and CVD mortality in stroke survivors. In addition, PA score, nicotine exposure score, and blood glucose score were significantly associated with all-cause and CVD mortality in stroke survivors. Of note, the addition of the depression score to the LE8 significantly improved the predictive value of all-cause mortality in stroke survivors, whereas it did not significantly improve the predictive value of CVD mortality.

The LC9 is a new assessment framework proposed with the addition of psychological health elements to the LE8, which aims to more comprehensively assess an individual’s CVH status. The close association of psychological well-being with cardiovascular risk and health has been widely established over the past decades, making it a basis for achieving optimal CVH (21). In a recently published prospective cohort study utilizing NHANES 2007–2018, Ge et al. (22) demonstrated that LC9 was associated with all-cause (HR of 0.80 per 10-point increase in LC9, 95% CI of 0.76–0.84, *p* < 0.001) and CVD mortality risk (HR of 0.78 per 10-point increase in LC9, 95% CI of 0.70–0.87, *p* < 0.001) in U.S. adults without CVD at baseline. The improvement in the predictive value of LC9 for all-cause and CVD mortality was significant compared with LE8 (all-cause mortality: NRI = 8.8%, IDI = 0.002; CVD mortality: NRI = 10.6%, IDI = 0.002) (22). Interestingly, our study demonstrated that adding the depression score to the LE8 significantly increased the ability to predict all-cause mortality after stroke (NRI, ΔC index, and IDI were all significantly improved, all *p* < 0.05). This means that approximately 10% of the study participants were reclassified into a more accurate risk category, which could have important implications for clinical decision-making and patient management. The ability to distinguish CVD mortality with the addition of the depression score had a similar but not statistically significant improvement. The NRI for CVD mortality, while not statistically significant, suggests a trend toward improved classification. Although the improvement is not statistically significant, it is still noteworthy and could be clinically meaningful in a larger sample or in a different population. For example, in a population of 1,000 stroke survivors, a 9–10% improvement in prediction could mean that 90–100 deaths are more accurately classified, which could have significant implications for clinical practice and public health. Even small improvements in prediction can have significant implications for patient care, especially in a high-risk population like stroke survivors. Our study demonstrated a significant and meaningful improvement in the predictive ability of LC9 for all-cause mortality in stroke survivors compared with LE8, but a similar degree but not statistically significant improvement in the predictive ability for CVD mortality.

Real-world studies on the association of CVH with the risk of mortality in stroke survivors are still relatively sparse. Ma et al. (23) similarly used data from NHANES 2005–2018 to suggest that LE8 was inversely associated with the risk of all-cause mortality in stroke survivors (HR and 95% CI of 0.85 [0.78, 0.94], *p* < 0.001). In a longitudinal cohort study from NHANES 2007–2018, Yan et al. (24) showed that compared to LE8 in the lowest tertile, stroke survivors in the highest tertile of LE8 had notably lower risks of all-cause mortality (HR 0.46, 95% CI 0.31–0.69, *p* < 0.001) and CVD mortality (HR 0.51, 95% CI 0.26–0.98, *p* = 0.043). A finding from the ARIC study demonstrated a significantly lower risk of all-cause mortality in stroke survivors with an LS7 of ≥10 points (compared with an LS7 of 4–6 points) in midlife (HR = 0.63, 95% CI 0.48–0.84) (29). Lin et al. (30) demonstrated in a cohort study from NHANES III (1988–1994) that stroke survivors meeting the 4+ CVH metrics had a 49% lower risk of all-cause mortality compared to a reference population meeting only 0–1 LS7 CVH metrics (HR = 0.51, 95% CI = 0.28–0.92; *p* for trend = 0.022). Our study demonstrated for the first time that LC9, which was recently proposed based on LE8, was inversely associated with the risk of both all-cause and CVD mortality in stroke survivors, further providing new evidence for adherence to high CVH for mortality prevention in stroke survivors.

Post-stroke depression is a well-established risk factor for mortality, and recent meta-analyses have shown that post-stroke depression is associated with worse functional outcomes and an approximately 60% increase in all-cause mortality in stroke survivors (31, 32). Our Cox regression analyses of the LC9 component scores showed inverse associations with all-cause and CVD mortality after stroke only for the PA score, nicotine exposure score, and blood glucose score, but not for the other scores including the depression score. We speculated that the discrepancies between these findings and previous studies stemmed primarily from the fact that there was a significant difference between the LC9 component scores and direct assessment of the metrics themselves (the former requires a specific score to be assigned based on the level of the metrics, whereas the latter does not). In addition, many of the methods of assessment of these CVH indicators in previous studies differ from our study. For example, PHQ-9 score assesses depressive symptoms by scoring continuously from 0 to 27, whereas the depression score in LC9 is assigned 0, 25, 50, 75, and 100 in descending order based on the range of PHQ-9 scores. In previous studies, post-stroke depression was assessed by a variety of other scales (e.g., Diagnostic and Statistical Manual of Mental Disorders, Center for Epidemiologic Studies Depression Scale, Hospital Anxiety and Depression Scale, and Hamilton Depression Rating Scale) or physician diagnosis, whereas the PHQ-9 was used in only one study (31). However, our results demonstrated that the addition of the depression score to the LE8 framework significantly improved the prediction of all-cause mortality in stroke survivors, underscoring the critical role of psychological health in CVH assessment. While the improvement in the predictive value of CVD mortality with the addition of the depression score was not statistically significant, it suggests a trend toward better classification. The non-significant improvement in the predictive value of CVD mortality with the addition of the depression score suggests that while psychological health is an important factor, its impact on CVD mortality may be more nuanced. The relatively small number of CVD mortality events in our study may have limited the statistical power to detect a significant improvement in prediction. Moreover, the mechanisms linking depression to CVD mortality may be more complex and less direct compared to the mechanisms linking depression to all-cause mortality. This could be due to the complex interplay between psychological health and other cardiovascular risk factors. Future studies should further explore the mechanisms underlying these relationships and consider larger sample sizes to better assess the clinical significance of these findings.

Our study has several strengths, including a nationally representative study design, a large sample and multiethnic study participants, and prospectively obtained mortality information. However, there are limitations to our study. First, the cross-sectional nature of the NHANES data means that we cannot establish causality between the LC9 score and mortality outcomes. The PHQ-9 was measured at a single time point, which may not fully capture the dynamic nature of depression over time. The nature of observational studies prevents these findings from being causally inferred and subject to residual confounding. Therefore, these findings need to be validated in future interventional studies. Future interventional studies are needed to explore the causal relationships between psychological health and mortality in stroke survivors. Interventions targeting psychological health, such as depression screening and treatment, could potentially improve cardiovascular outcomes and reduce mortality. These studies should determine the effectiveness of such interventions in improving both psychological and CVH in stroke survivors. The assessment of LC9 and stroke was largely based on self-report and therefore may be affected by recall bias. Information on stroke severity, stroke subtype, and lesion location was not available in NHANES, and thus the impact of these important factors could not be explored. Stroke subtypes and post-stroke rehabilitation outcomes may have potential effects on these associations. However, due to limitations of the NHANES data, we were unable to assess the impact of these factors. The impact of these factors needs to be clarified in subsequent studies. Whether these findings can be generalized to in-hospital stroke patients and other national/ethnic populations requires further study. In addition, the effect of LC9 on short-term mortality in stroke survivors needs to be further explored. Another important limitation is that the psychological health component of LC9 should include factors such as depression, anxiety disorders, chronic and traumatic stress, and social integration. Psychological health is a multifaceted construct that includes not only depression but also other factors such as anxiety, stress, social integration, and chronic trauma. These factors can have significant impacts on CVH and mortality. There were significant associations between psychological functioning and CVD, and other negative and positive psychological effects have not been included/examined (33). Masters et al. (33) highlighted the broad impact of psychological functioning on CVD, emphasizing that other factors such as anxiety, negative affectivity, and Type D personality can contribute to adverse health outcomes. Moreover, chronic stress has been shown to increase blood pressure and inflammation (34), while social isolation has been linked to higher rates of cardiovascular mortality (35). Due to NHANES data limitations, we only assessed depression, which may have misestimated the effect size of the association. Psychological health is multidimensional, and although the optimal integration of psychological fitness metrics with CVH is still uncertain, further research is necessary to identify the most influential measures and to improve the predictive validity of comprehensive psychological health evaluations for LC9. Future exploration of improvements in the predictive power of comprehensive psychological health assessments for LC9 is warranted.

## 5 Conclusion

Our study conclusively demonstrates an inverse association, with a dose–response relationship, between LC9 and all-cause as well as CVD mortality among stroke survivors. Additionally, significant inverse associations were also found for PA score, nicotine exposure score, and blood glucose score. Compared with LE8, LC9 shows a significant improvement in predictive value for all-cause mortality in stroke survivors, while for CVD mortality it shows a trend toward improved classification although not clinically significant. Adding depression score assessment to the existing LE8 framework may improve the prediction of all-cause mortality in stroke survivors.

## Data Availability

Publicly available datasets were analyzed in this study. This data can be found: https://www.cdc.gov/nchs/nhanes/.
